# Effects of Nitrogen Addition on Litter Decomposition and CO_2_ Release: Considering Changes in Litter Quantity

**DOI:** 10.1371/journal.pone.0144665

**Published:** 2015-12-11

**Authors:** Hui-Chao Li, Ya-Lin Hu, Rong Mao, Qiong Zhao, De-Hui Zeng

**Affiliations:** 1 State Key Laboratory of Forest and Soil Ecology, Institute of Applied Ecology, Chinese Academy of Sciences, Shenyang, China; 2 University of Chinese Academy of Sciences, Beijing, China; 3 Northeast Institute of Geography and Agroecology, Chinese Academy of Sciences, Changchun, China; National Taiwan University, TAIWAN

## Abstract

This study aims to evaluate the impacts of changes in litter quantity under simulated N deposition on litter decomposition, CO_2_ release, and soil C loss potential in a larch plantation in Northeast China. We conducted a laboratory incubation experiment using soil and litter collected from control and N addition (100 kg ha^−1^ year^−1^ for 10 years) plots. Different quantities of litter (0, 1, 2 and 4 g) were placed on 150 g soils collected from the same plots and incubated in microcosms for 270 days. We found that increased litter input strongly stimulated litter decomposition rate and CO_2_ release in both control and N fertilization microcosms, though reduced soil microbial biomass C (MBC) and dissolved inorganic N (DIN) concentration. Carbon input (C loss from litter decomposition) and carbon output (the cumulative C loss due to respiration) elevated with increasing litter input in both control and N fertilization microcosms. However, soil C loss potentials (C output–C input) reduced by 62% in control microcosms and 111% in N fertilization microcosms when litter addition increased from 1 g to 4 g, respectively. Our results indicated that increased litter input had a potential to suppress soil organic C loss especially for N addition plots.

## Introduction

The reactive nitrogen (N) deposition has increased from 32 Mt N year^-1^ to approximately 116 Mt N year^-1^ since 1860 at global scale and it is expected a further enhancement in future [1, 2], due to human activities such as fertilizer application, fossil fuel combustion and legume cultivation [3]. Increased N deposition can dramatically alter soil N availability and N cycling [4, 5], litter quantity and quality and soil physicochemical environment [6], and thus affect litter decomposition processes in terrestrial ecosystems [7, 8]. It is important to understand impacts of N deposition on carbon (C) flux of ecosystems [6, 9], since soil organic C (SOC) dynamics is strongly linked to soil N dynamics.

Effects of N deposition on soil C cycling have been widely studied, but the results are still highly inconsistent [10]. Plant litter decomposition and soil respiration are two major interdependent processes regulating the global terrestrial C cycling. Most previous studies revealed that N addition suppressed soil respiration and thus sequestered soil C in terrestrial ecosystems, through a shift in organic matter chemistry and biomass and composition of soil microbial communities [11, 12]. However, the effects of N addition on litter decomposition are more variable with a stimulation [13, 14] or inhibition [15, 16], particularly in forest ecosystems. A meta-analysis by Knorr et al. [17] found that the effect of N addition on litter decomposition depends on plant (tree) species and litter quality. Therefore, clarifying the complex interactions between soil C loss through respiration and litter C input into soil during litter decomposition may help us to better understand the responses of soil C sequestration to N addition in terrestrial ecosystems [18].

Increased N input has been shown to enhance plant productivity and aboveground biomass [19, 20] due to the N limitation of plant growth worldwide. Liu and Greaver [6] synthesized data from global ecosystems and found that N addition increased aboveground litter input by 20%. Changes in litter input rate could alter litter decomposition and CO_2_ release, and thus affect soil C storage. A recent study by Chen et al. [21] showed that litter decomposition rates elevated with increasing amount of litter input in a tropical forest ecosystem. They observed decreased N and P reaming in litter-added plots compared to control plots indicating that increased substrate availability could stimulate the microbes to release litter nutrients, which partially contribute to increased decomposition of leaf litter [21]. In addition, change of microbial biomass and microbial communities in litter layer after increasing litter input, such as an increased ratio of fungal to bacterial abundance in litter layer, could result in increased mass loss of leaf litter [22]. Giardina et al. [23] found that increased aboveground litter input accelerated soil CO_2_ release and decreased soil C storage due to priming effect [24–26]. Though litter quantity has been found to have a significant influence on C cycling, no studies directly investigated the effect of changes in litter quantities under N addition on litter decomposition and soil C mineralization. Therefore, studies of changes in litter inputs, as a response to elevated N deposition, are indispensable to making accurate predictions of the dynamics of forest ecosystem structure and function in future.

Dahurian larch (*Larix gmelinii* Rupr.) is a most common commercial tree species in Northeast China. Given ever-increasing N deposition and resultant ecological consequences in China [4], a long-term N addition experiment was started in 2002 in a Dahurian larch plantation in Northeast China. It offers a unique opportunity to examine how combined effects of N addition and litter quantity may affect litter decomposition and C and nutrient cycling. The effects of N addition on soil chemical and biological properties have been investigated in the larch plantations [27, 28]. Jia et al. [28] found that N fertilization decreased soil microbial biomass C (MBC) and N (MBN) by 29% and 42% in the larch plantations, respectively. In addition, we found that chronic N addition increased larch leaf litter production by 14% (data not shown), which may result in a significant increase of C input into soil. Higher C input to soil could result in priming effect on soil respiration [24–26], because soil microbes are usually C limited. In this study, a microcosm incubation experiment was conducted to examine the effects of increased litter quantity on litter decomposition and CO_2_ release rate under N addition. We hypothesized that: (1) the increased amount of litter input would increase litter decomposition rate and CO_2_ release rate due to alleviating C limitation to microbial growth; while (2) litter decomposition rate and CO_2_ release rate would be lower in N fertilization microcosms due to the lower microbial biomass compared to the control microcosms.

## Materials and Methods

### Study site and N addition experiment

Soil and leaf litter used for the incubation experiment were collected from a long-term N addition experiment site located at the Maoershan Forest Ecosystem Research Station (45°21′–45°25′N, 127°30′–127°34′E) of Northeast Forestry University in Heilongjiang, China. Mean annual air temperature in this site is 2.8°C, ranging from –19.6°C in January to 20.9°C in July. Mean annual precipitation is 723 mm [29]. Soil is classed as a well-drained Hap-Boric Luvisol [30].

This N addition experiment has been carried out since 2002. The Dahurian larch plantation was established in 1986 by planting nursery-raised 2-year-old bare root seedlings with a space of 1.5 m × 2.0 m. Six 20 m × 30 m plots were established, and there is a 10-m buffer strip between adjacent plots. Two N fertilization treatments (control and N addition) with three replicates were randomly arranged in these plots. Nitrogen has been added as ammonium nitrate at a rate of 100 kg N ha^−1^ year^−1^ in pellets monthly from May to September since 2002. This N addition was chosen is to simulate anthropogenic N deposition.

### Soil and litter collection

Collecting samples in field has got permission from Maoershan Forest Ecosystem Research Station of Northeast Forestry University. For our incubation experiment, leaf litter and soil were collected from the control and N addition plots in October 2012. We collected top soil (0–10 cm) with a soil auger (10 cm in diameter) after removing the surface litter layer. At the same time, we harvested naturally senesced larch leaf litter using six nylon nets (1 m × 1 m; 2-mm mesh) placed randomly in each plot and placed at a height of 80 cm aboveground in late September when massive leaf litter fall began. Soil samples were sieved (<2 mm) to remove roots and plant detritus. Soil and leaf litter samples were divided into two sub-samples, respectively. One sub-sample of soil and litter collected from each plot was oven-dried to a constant mass at 60°C and was used to analyze the chemical characteristics of soil and leaf litter after 10-year N addition. Another sub-sample of soil and litter was composited by treatment separately, and the soil was stored at 4°C, and litter was air-dried at room temperature until the litter decomposition experiment started.

### Litter decomposition experiment

For litter incubation experiment, fresh soil (equivalent to 150 g dry mass) collected from the control or N addition plots was placed into a plastic jar (10 cm in diameter, 12 cm tall). Then leaf litter collected from the corresponding control or N addition plots was placed on the surface of soil at rates of 0, 1.00, 2.00, 4.00 g, respectively. The water content of air-dried leaf litter used for incubation was 11%. Specifically, the addition rate of 2-g litter was equal to annual amount of leaf litterfall in control plots. In total, eight treatments were set up with five replicates: soil + 0 g litter from control plots (CL0), soil + 1 g litter from control plots (CL1), soil +2 g litter from control plots (CL2), soil + 4 g litter from control plots (CL4), soil + 0 g litter from N addition plots (NL0), soil + 1 g litter from N addition plots (NL1), soil + 2 g litter from N addition plots (NL2), soil + 4 g litter from N addition plots (NL4).

All microcosms were incubated at 25°C in dark conditions in an incubator. Water was added every three days to keep 60% of soil water-holding capacity during incubation by weighing method. During incubation, a perforated adhesive film was placed on the opening of plastic jars in order to reduce water losses while allowing gaseous exchange.

### Determination of CO_2_ release rate in microcosms

CO_2_ release rates were measured at 7, 14, 21, 28, 42, 70, 119, 180, 270 days of incubation. The plastic jars were completely flushed with fresh air before gas sampling, and then sealed with a lid with a septum at 25°C in dark conditions in incubator. After 45 min, the headspace gas (35 mL) was sampled using a syringe and CO_2_ concentration (ppm) was analyzed by gas chromatography (GC-7890A, Agilent, USA) with an electron capture detector (ECD). The conversion equation is based on 25°C and 1 atmosphere: CO_2_ concentration (mg CO_2_ d^-1^ kg^-1^ soil) = 44/22.4 × CO_2_ concentration (ppm) × 273/(273+25)/24*t*×*V*/*m*/10^3^, where *t* is the incubation time (h) before gas sampling, *V* is gas volume (m^3^) in plastic jars and *m* is soil dry weight (g).

### Litter and soil sampling and chemical analysis

Litter and soil samples were destructively collected at 14, 28, 42, 70, 119, 180 and 270 days from five microcosms of each treatment with a total of 40 jars (8 treatments × 5 replications) for each sampling time. Litter samples were dried at 60°C for 48 h, weighed and then ground to pass through 0.25 mm sieve for analysis of C and N concentrations. Soil samples were stored at 4°C for no longer than 2 days before determination of MBC, MBN, DOC and DIN (NO_3_^-^-N and NH_4_^+^-N).

Organic C concentration was measured by the Walkey and Black K_2_Cr_2_O_7_–H_2_SO_4_ oxidation method [31]. Total N and P concentrations were determined using a continuous-flow autoanalyzer (AutoAnalyzer III, Bran+Luebbe GmbH, Germany) after digestion in 5 mL H_2_SO_4_ with a catalyst (mixture of CuSO_4_ and K_2_SO_4_) [32]. We measured lignin using a modified acetyl bromide method with samples calibrated against a standard of lignin (lignin, alkali, 2-hydroxypropyl ether) [33]. Cellulose concentration was determined using a modified acid-hydrolysis method by Updegraff [34]. Fifty-milligram litter sub-sample was extracted by the solution of CH_3_COOH and HNO_3_ in boiling water bath for 25 min. The reaction was cooled and the tubes were centrifuged at 13,000 rpm for 5 min. After the supernatant was removed, 10% H_2_SO_4_ and anthrone reagent were added into the tubes. The concentration of cellulose was measured at 620 nm. Total polyphenols were extracted with 70% methanol and then measured colorimetrically using the Folin-Ciocalteu method [35].

Soil MBC and MBN were determined by the chloroform fumigation extraction method [36]. For each soil sample, six sub-samples (equal to 10-g dry mass) were weighed into beakers. Three sub-samples were fumigated with chloroform for 24 h, and then were extracted with 100 mL 0.5 M K_2_SO_4_. Soil MBC and MBN were calculated by the differences of organic C and N concentration in the solution between the fumigated and non-fumigated soils with an extraction coefficient of 0.38 for MBC and 0.45 for MBN, respectively [37]. The concentration of organic C in the 0.5 M K_2_SO_4_ extract solution of the non-fumigated soil was used as a proxy for DOC in the soil [38]. For determination of soil NO_3_^-^-N and NH_4_^+^-N concentrations, 10-g soil (dry mass) was extracted by 50 mL 2 M KCl solution [39], and then analyzed with a continuous-flow autoanalyzer (AutoAnalyzer III, Bran + Luebbe GmbH, Germany).

### Calculations and statistical analyses

To assess net N dynamics of decomposing litter, the litter N remaining was expressed as percentage of N content of decomposing litter to the initial content. Litter N contents were calculated by multiplying N concentration of litter (mg g^−1^) by litter mass remaining (g) during incubation period. Cumulative CO_2_ release (g) after 270-day incubation was integrated using trapezoidal rule by Origin (version 8.5, OriginLab Corporation).

Litter decomposition constants (*k*) were estimated by a linear regression after logarithmic transformation as follows: ln (*W*_*t*_/*W*_0_) = –*kt*, where *W*_*t*_ is the remaining litter dry mass at time *t*, *W*_0_ is the initial dry mass. Regression lines were compared by analysis of covariance (ANCOVA) followed by a Tukey’s multiple-comparison test.

In addition, to better understand the effects of litter addition on soil C turnover during incubation, we identified ‘soil C loss potential’ by calculating the value of C output minus C input, where C output is cumulative C loss due to litter and soil respiration, and C input is transferred from mass loss of litter.

The differences of initial chemistry of litter and soil used in the incubation between the control and N addition were analyzed by one-way ANOVA. Effects of litter addition rate and N fertilization on the decomposition constant (*k*), total litter mass loss, litter N remaining and cumulative CO_2_-C release were analyzed by split-plot ANOVA. Then we analyzed the effects of litter addition rate on *k*, total litter mass loss and litter N remaining and cumulative CO_2_-C release in control and N fertilization microcosms separately, and the effects of N addition on k and cumulative CO_2_-C release for each litter addition rate separately by one-way ANOVA. Effects of litter addition rate and N addition on CO_2_ release rate and soil MBC, MBN, DOC and DIN were analyzed using repeated measures ANOVA. When there were significant interactions between litter addition rate and N addition, one-way ANOVA was used to test the effects of litter addition rate on CO_2_ release rate, soil MBC, MBN, DOC and DIN in control and N fertilization microcosms separately, and the effects of N addition on CO_2_ release rate and soil MBC, MBN, DOC and DIN for each litter addition rate separately. The Tukey’s multiple-comparison test was used to compare the means. All data were statistically analyzed using SPSS PASW Statistics (version 18.0, SPSS Inc.). The significant level was at *α* = 0.05.

## Results

### Initial chemical characteristics of soil and leaf litter

Litter total N concentration increased by 8.8% in the N addition plots as compared with the control plots (*P* = 0.002), but no differences were found for litter total C, total P, cellulose, lignin and polyphenol concentrations (Table 1). Litter C:N ratio was lower in the N addition plots than in the control plots (*P* = 0.004), due to significantly increased litter N concentration. Soil organic C and total N concentrations increased significantly after 10-year N addition (*P*<0.001 and *P* = 0.043, respectively).

**Table 1:** Chemical characteristic of leaf litter and soil after 10 years of N addition in a larch plantation (mg g^-1^).

	N addition	Control	*P* values
***Litter***			
**Cellulose**	365.29 ± 9.09	355.45 ± 7.71	0.433
**Lignin**	348.22 ± 28.71	353.05 ± 16.49	0.817
**Polyphenol**	11.74 ± 0.43	11.06 ± 0.59	0.373
**Organic C**	466.40 ± 3.20	468.93 ± 4.49	0.661
**Total N**	5.66 ± 0.09	5.20 ± 0.05	0.002
**Total P**	0.37 ± 0.06	0.38 ± 0.08	0.104
**C:N**	82.50 ± 1.07	90.29 ± 1.63	0.004
***Soil***			
**Organic C**	119.31 ± 2.71	109.64 ± 1.81	0.000
**Total N**	6.00 ± 0.36	5.53 ± 0.23	0.043
**Total P**	1.30 ± 0.10	1.32 ± 0.10	0.108

The difference of means between control and N addition was determined by one-way ANOVA. Values are means ± SE (*n* = 3).

### Litter mass loss and decomposition constant

**Fig 1:**
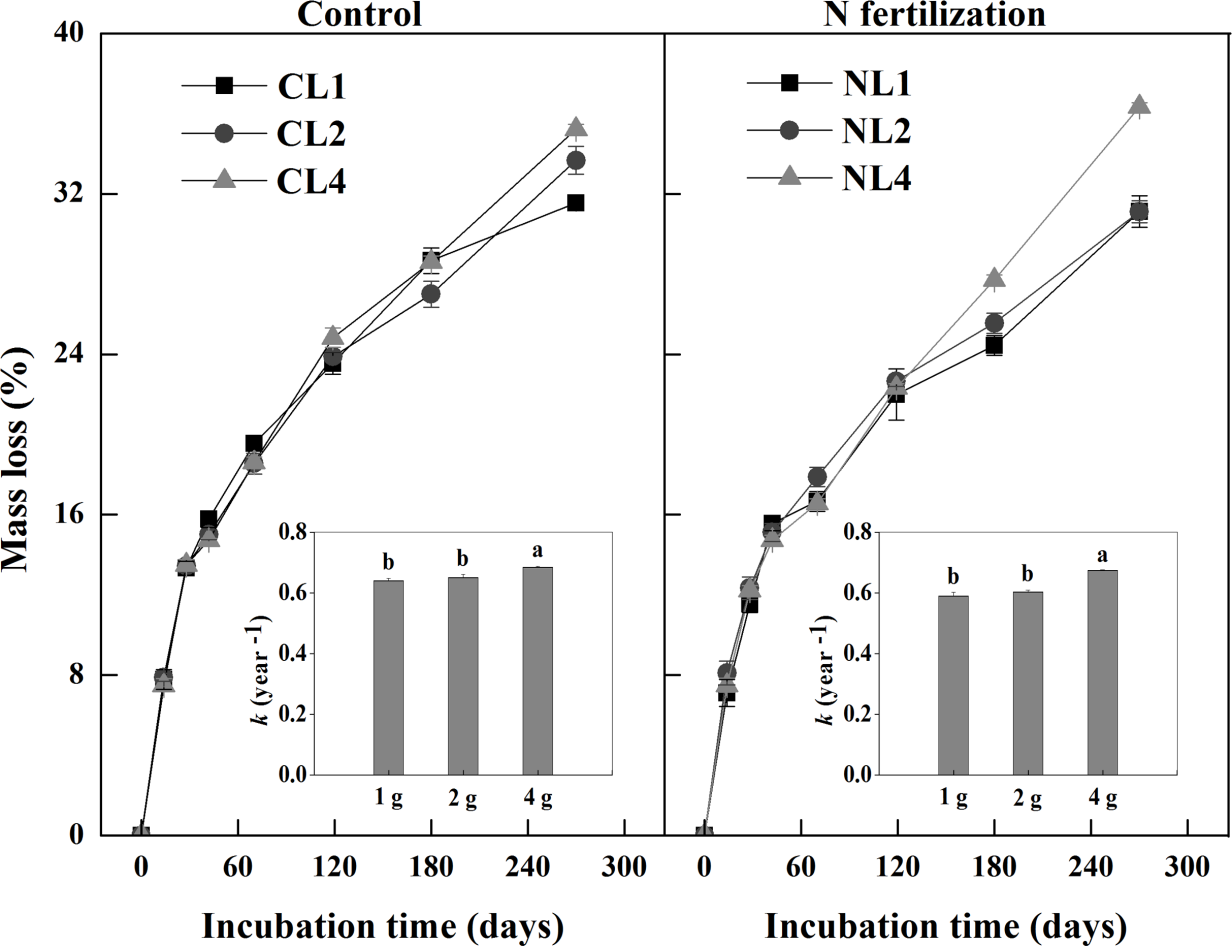
Mass loss and decomposition constant *k* (the insets) of leaf litter among different input quantity treatments during incubation in the control and N fertilization microcosms. Values are means (*n* = 5) ± SE. Different letters indicate significant differences (*P*<0.05) among treatments. *CL1* soil + 1 g litter from control plots, *CL2* soil +2 g litter from control plots, *CL4* soil + 4 g litter from control plots, *NL1* soil + 1 g litter from N addition plots, *NL2* soil + 2 g litter from N addition plots, *NL4* soil + 4 g litter from N addition plots.

There was a significant interaction of N fertilization and litter addition rate on total litter mass loss after 270 days of incubation (*P* = 0.009). There was no difference of total litter mass loss between the control and N fertilization microcosms (*P* = 0.159). Litter mass loss elevated with increasing litter input in both the control and N fertilization microcosms (both *P*<0.001; Table 2). Specifically, C loss from litter decomposition (as C input into soil) ranged from 0.17 g C in the CL1 to 0.75 g C in the CL4 and from 0.18 g C in the NL1 to 0.76 g C in the NL4 (data were calculated on the basis of litter mass loss; Fig 1).

**Table 2:** Changes in litter mass loss, litter N remaining and cumulative CO_2_-C release of the control and N fertilization microcosms under different litter input quantity treatments after 270 days of incubation.

Microcosms	Litter addition	Mass loss	Litter N remaining	Cumulative CO_2_-C release
		(%)	(%)	(g C)
Control	0 g			0.58 ± 0.03b
	1 g	31.56 ± 0.27c	138.83 ± 3.58a	0.64 ± 0.04b
	2 g	33.67 ± 0.69b	134.32 ± 0.99ab	0.67 ± 0.01b
	4 g	35.22 ± 0.24a	124.04 ± 3.67b	0.93 ± 0.04a
N fertilization	0 g			0.39 ± 0.02c
	1 g	31.11 ± 0.79b	128.19 ± 5.18a	0.53 ± 0.02b
	2 g	31.11 ± 0.56b	127.96 ± 1.67a	0.69 ± 0.02a
	4 g	36.33 ± 0.20a	113.83 ± 4.24b	0.71 ± 0.02a
*P* value	**N**	0.159	0.077	0.552
	**A**	<0.001	<0.001	0.001
	**N×A**	0.009	0.683	0.001

Values are means ± SE (*n* = 5). Effects of N addition (N) and litter addition rate (A) were determined by a split-plot ANOVA. Letters denote significant differences among means within a column.

There was a significant interaction of N fertilization and litter addition rate on litter decomposition constant (*k*) (*P* = 0.039). Litter decomposition constants were higher in NL4 than in NL1 and NL2 (*P*<0.001; Fig 1). Similarly, litter decomposition constant was higher in CL4 than in CL1 and CL2 (*P* = 0.006). Litter decomposition constant was lower in NL1 than in CL1, and was lower in NL2 than in CL2 (Fig 1).

### Litter N dynamics

**Fig 2:**
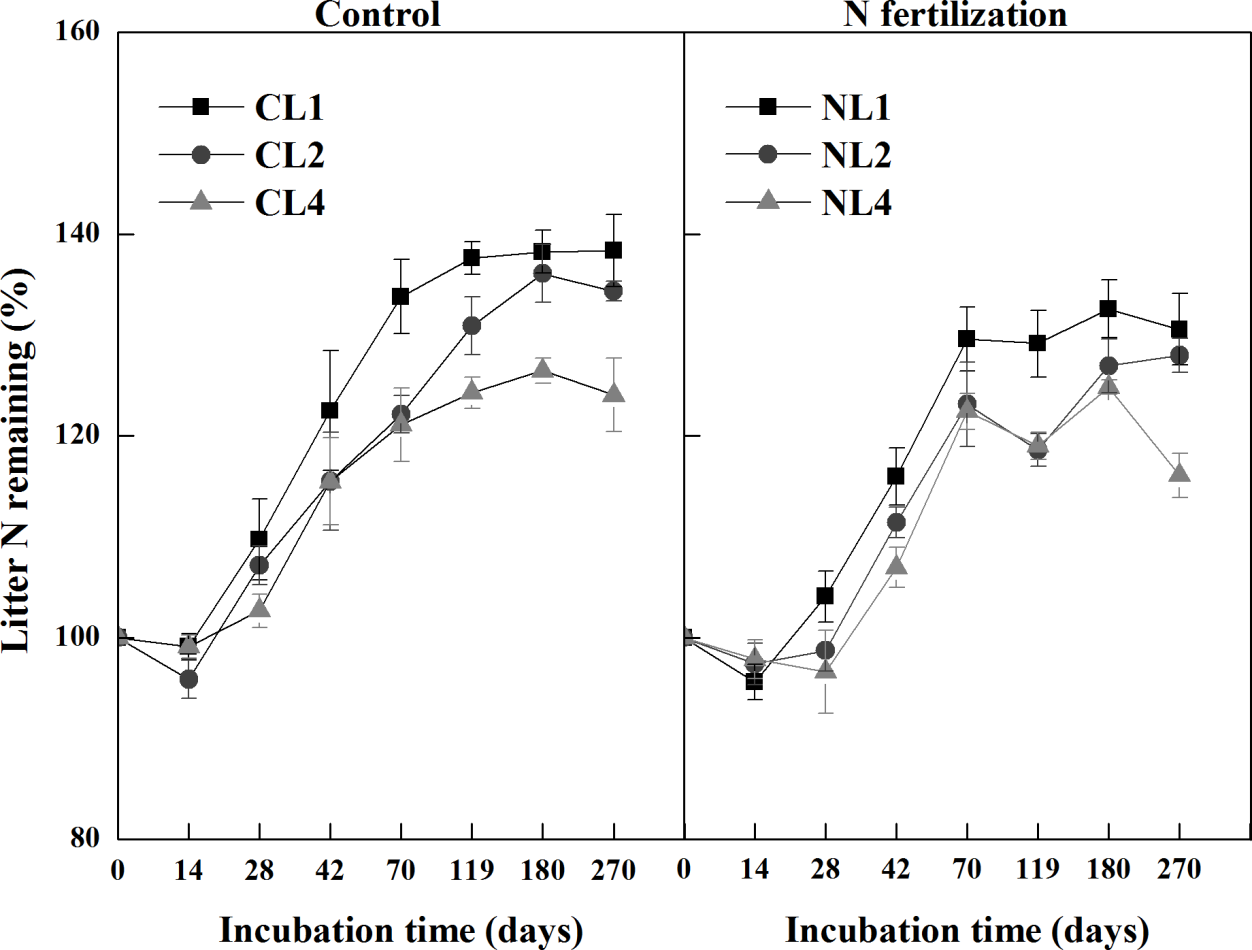
Percent N remaining of leaf litter among different litter input quantity treatments during incubation in the control and N fertilization microcosms. Values are means (*n* = 5) ± SE. *CL1* soil + 1 g litter from control plots, *CL2* soil +2 g litter from control plots, *CL4* soil + 4 g litter from control plots, *NL1* soil + 1 g litter from N addition plots, *NL2* soil + 2 g litter from N addition plots, *NL4* soil + 4 g litter from N addition plots.

Leaf litter N showed a net immobilization after transient release in all six litter-amended treatments during the 270 days of incubation (Fig 2). There was no significant interaction of N fertilization and litter addition rate on litter N remaining (*P* = 0.683). Litter N remaining was lower in the N fertilization microcosms than in the control microcosms (*P* = 0.020; Fig 2). For both the control and N fertilization microcosms, litter N remaining generally decreased with increasing litter addition (*P* = 0.016 and *P* = 0.040, respectively).

### CO_2_ release rates

**Fig 3:**
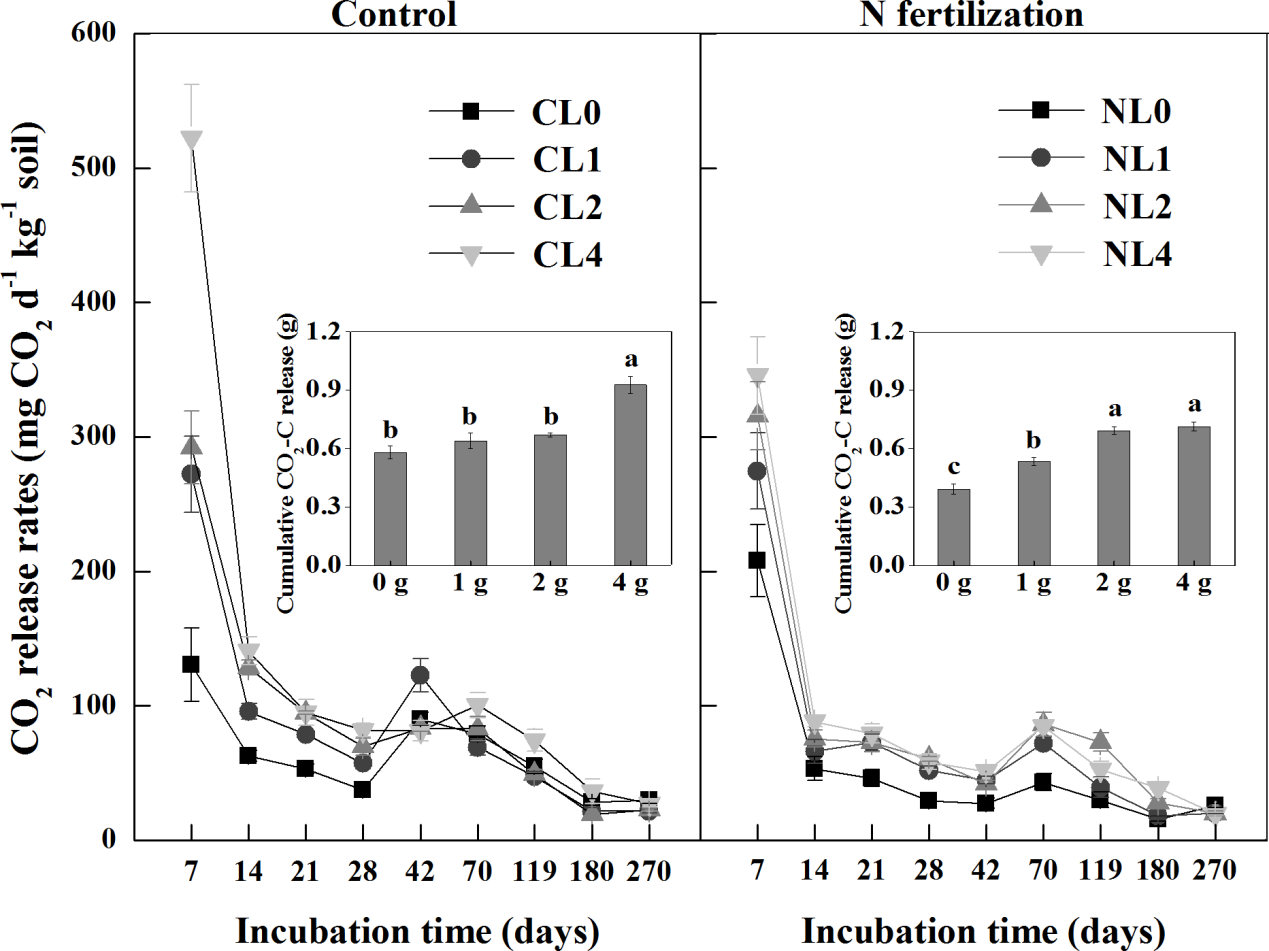
CO_2_ release rates and cumulative CO_2_-C release (the insets) among different litter input quantity treatments during incubation in the control and N fertilization microcosms. Values are means (*n* = 5) ± SE. *CL0* soil + 0 g litter from control plots, *CL1* soil + 1 g litter from control plots, *CL2* soil +2 g litter from control plots, *CL4* soil + 4 g litter from control plots, *NL0* soil + 0 g litter from N addition plots, *NL1* soil + 1 g litter from N addition plots, *NL2* soil + 2 g litter from N addition plots, *NL4* soil + 4 g litter from N addition plots.

The rates of CO_2_ release showed a similar temporal pattern among different litter input treatments with a sharp decline in the first 14 days and then smoothly proceeded over time in both the control and N fertilization microcosms (Fig 3). There was a significant interaction of N fertilization and litter addition rate on CO_2_ release rates and cumulative CO_2_ release (*P* = 0.017 and *P* = 0.001, respectively). In general, N fertilization significantly inhibited CO_2_ release rates, with a lower cumulative CO_2_ release (*P*<0.001; Table 2; Fig 3). Carbon dioxide release rate was higher in CL4 than in NL4 (*P*<0.001; Fig 3), but there were no differences between CL0 and NL0, between CL1 and NL1 and between CL2 and NL2. The higher litter addition increased CO_2_ release in both the control and N fertilization microcosms (all *P*<0.001; Fig 3). The cumulative CO_2_ loss in CL4 increased by 60% as compared with CL0 (Fig 3). Similarly, NL1, NL2 and NL4 increased cumulative CO_2_ release by 36%, 77% and 82% compared with NL0, respectively (Fig 3). Specifically, the cumulative C loss due to litter and soil respiration (C output) ranged from 0.64 g C in the CL1 to 0.93 g C in the CL4 and from 0.53 g C in the NL1 to 0.71 g C in the NL4 (Fig 3). Soil C loss potentials (C output–C input) were 0.47 in CL1 and 0.35 in NL1, 0.32 in CL2 and 0.34 in NL2, and 0.18 in CL4 and -0.05 in NL4, respectively.

### MBC, MBN, DOC and DIN

**Fig 4:**
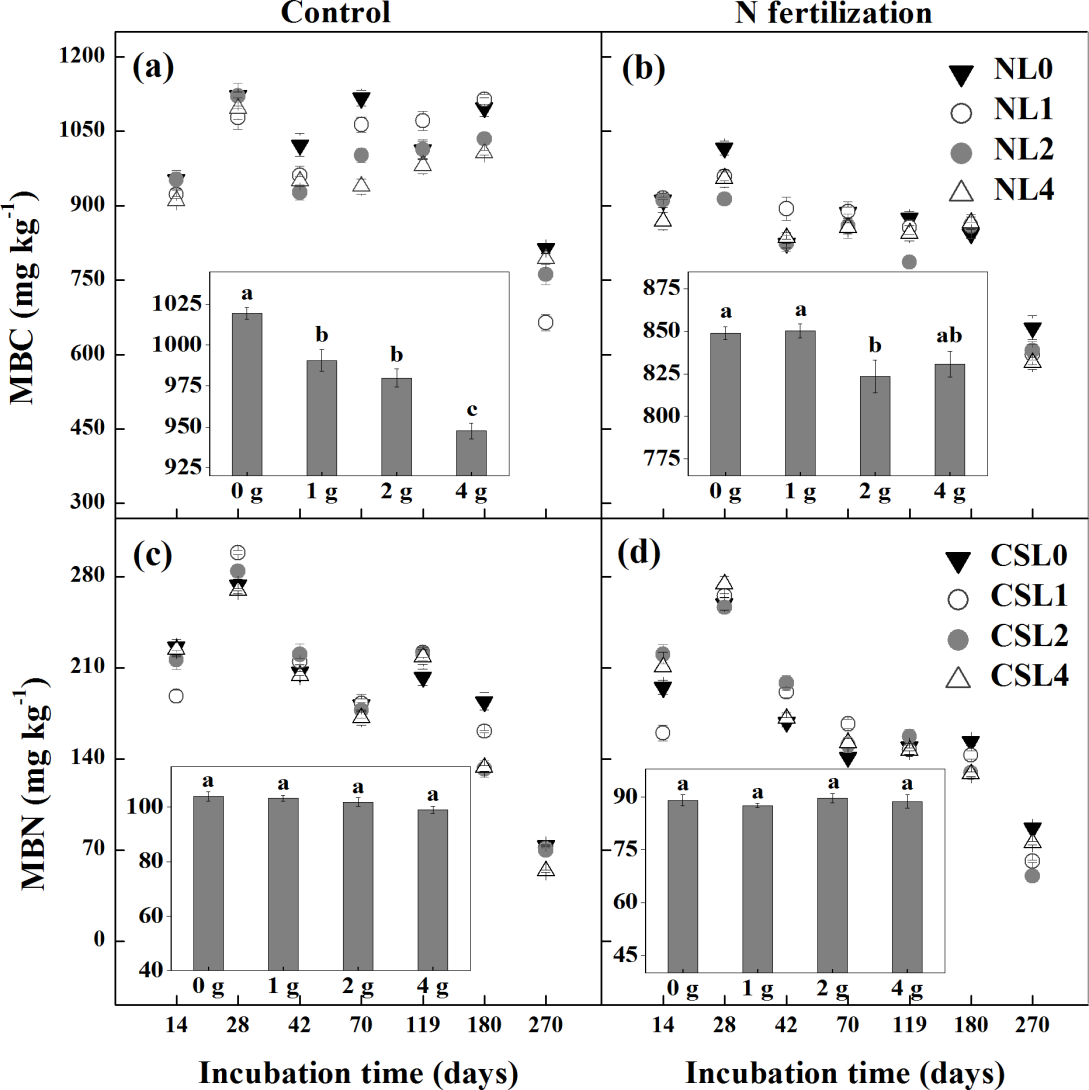
Soil microbial biomass C (MBC; a and b) and microbial biomass N (MBN; c and d) among different litter input quantity treatments during incubation in the control and N fertilization microcosms. Values are means (*n* = 5) ± SE. *CL0* soil + 0 g litter from control plots, *CL1* soil + 1 g litter from control plots, *CL2* soil +2 g litter from control plots, *CL4* soil + 4 g litter from control plots, *NL0* soil + 0 g litter from N addition plots, *NL1* soil + 1 g litter from N addition plots, *NL2* soil + 2 g litter from N addition plots, *NL4* soil + 4 g litter from N addition plots. In the insets, the means of MBC or MBN among different litter input treatments during the whole incubation time in the control and N fertilization microcosms are shown (*n* = 7).

Soil MBC and MBN of the control and N fertilization microcosms showed a similar temporal pattern with a peak occurring at day 28, and then smoothly proceeded over time until a fast decrease at day 270 (Fig 4). There was a significant interaction of N fertilization and litter addition rate on MBC (*P* = 0.001), but not on MBN (*P* = 0.119). In general, soil MBC and MBN were lower in the N fertilization microcosms than in the control microcosms (*P*<0.001; Fig 4). MBC was higher in CL0 than in CL1, CL2 and CL4, and higher in CL2 and CL1 than in CL4 (*P*<0.001; Fig 4A), but there was no difference between CL2 and CL1. Similarly, MBC was lower in NL2 than in NL0 and NL1 (*P*<0.001; Fig 4B), but there were no differences among NL0, NL1 and NL4 and between NL2 and NL4. Litter addition rate had no significant effect on soil MBN (*P* = 0.119; Fig 4C and 4D).

**Fig 5:**
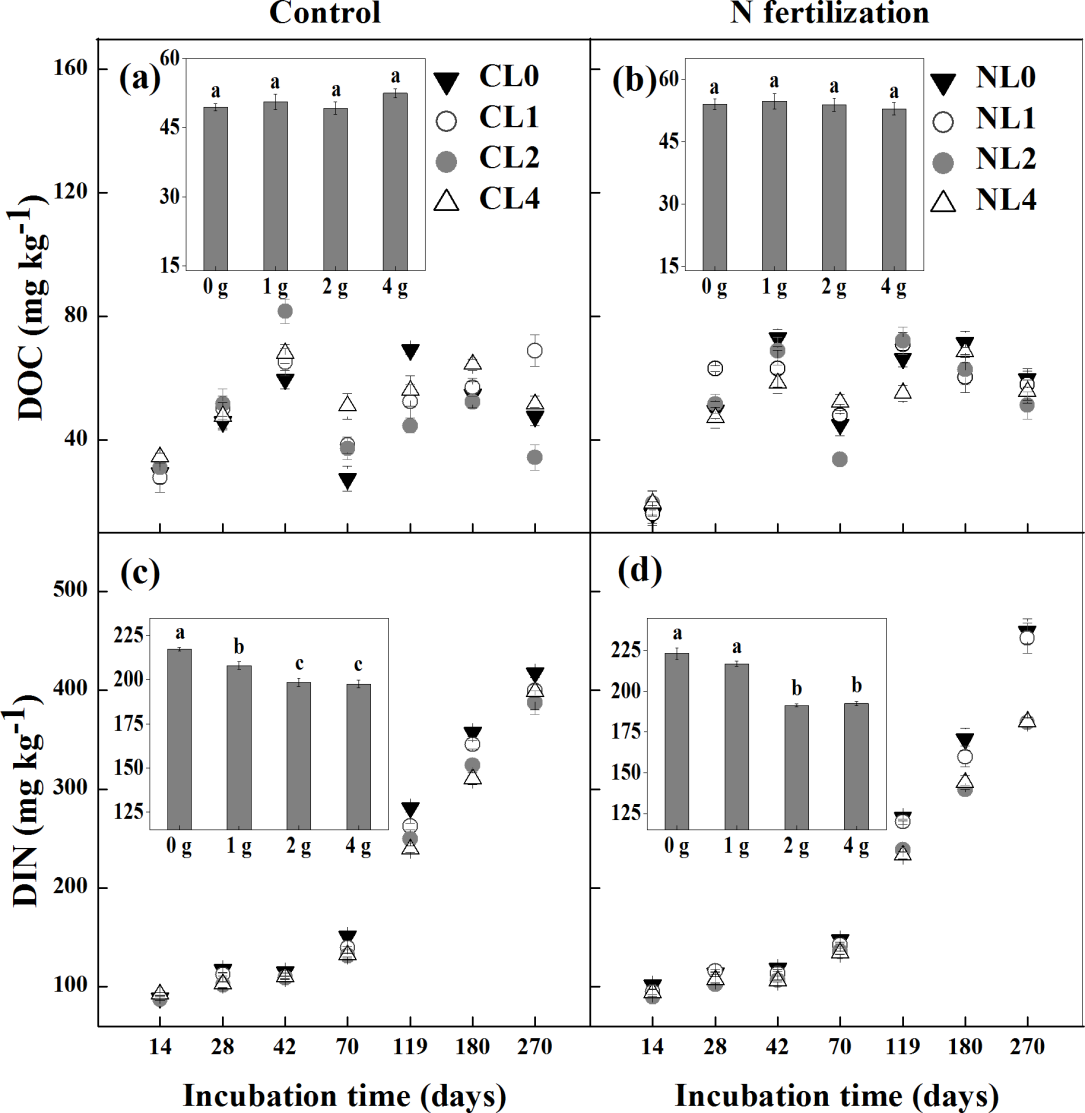
Soil dissolved organic C (DOC; a and b) and dissolved inorganic N (DIN; c and d) among different litter input quantity treatments during incubation in the control and N fertilization microcosms. Values are means (*n* = 5) ± SE. *CL0* soil + 0 g litter from control plots, *CL1* soil + 1 g litter from control plots, *CL2* soil +2 g litter from control plots, *CL4* soil + 4 g litter from control plots, *NL0* soil + 0 g litter from N addition plots, *NL1* soil + 1 g litter from N addition plots, *NL2* soil + 2 g litter from N addition plots, *NL4* soil + 4 g litter from N addition plots. In the insets, the means of DOC or DIN among different litter input treatments during the whole incubation time in the control and N fertilization microcosms are shown (*n* = 7).

There was no significant interaction of N fertilization and litter addition rate on DOC (*P* = 0.085). DOC was higher in NL0 than in CL0 (*P*<0.001; Fig 5A and 5B), but there were no differences between CL1 and NL1, between CL2 and NL2 and between CL4 and NL4. Litter addition rate had no significant effect on DOC (*P* = 0.345; Fig 5A and 5B).

Soil DIN consistently increased over the period of incubation under both the control and N addition (Fig 5C and 5D). Compared with non-litter-amended soil (CL0 and NL0), litter addition significantly decreased soil DIN in the control and N fertilization microcosms after 28 days, respectively (all *P*<0.001; Fig 5C and 5D). A significant interaction of N fertilization and litter addition rate on DIN was observed (*P*<0.001). DIN was higher in NL1 than in CL1 (*P*<0.001; Fig 5C and 5D), but lower in NL2 than in CL2 (*P*<0.001; Fig 5C and 5D). There were no differences between CL0 and NL0 and between CL4 and NL4. DIN was higher in CL0 than in CL1, CL2 and CL4, and higher in CL1 than in CL2 and CL4 (*P*<0.001; Fig 5C and 5D), but there was no difference between CL2 and CL4. Similarly, DIN was higher in NL0 and NL1 than in NL2 and NL4 (*P*<0.001; Fig 5C and 5D), but there were no differences between NL0 and NL1 and between NL2 and NL4.

## Discussion

### Effect of litter addition rate

We found that increased litter input strongly stimulated litter decomposition rate and CO_2_ release, which is consistent with what has already been reported by Liu et al. [40] who found that the cumulative C loss increased with increasing litter addition. However, increased litter input did not increase MBC (Fig 4). Instead, microbial C:N ratios significantly decreased in higher litter addition rate. Our result indirectly supports Buchkowski et al. [41], who showed that microbial biomass was assumed to play an important role in controlling C mineralization rates only when C:N ratios of the litter inputs were close to those of the microbial biomass. Otherwise, microbial stoichiometry is likely to be the primary driver of C mineralization rate. Indeed, the C:N ratios of the litter in the control and N fertilization microcosms (90.3 and 82.5) were much higher than those of the microbial biomass (9.6 and 11.6) in our study. The reason of microbial stoichiometry as a regulator of soil C cycling may be due to stoichiometric limitations of the microbial pool [42, 43]. In our study, for example, litter addition increased the relative C availability for microbes. To offset the elevated C availability and maintain the relative stability of microbial stoichiometry, microbial community increased respiration to waste more C. In our study, litter mass loss (Fig 1) and C output through respiration (Fig 3) elevated with increasing litter input in both the control and N fertilization microcosms. At the same litter addition rate, we found that C output through respiration was higher than C input from litter decomposition (data were calculated on the basis of litter mass loss; Fig 1) across all microcosms. However, soil C loss potential across the N addition and control microcosms decreased with increasing litter addition, implying that higher litter input has a potential to decrease soil C loss relatively.

Fontaine et al. [24] suggested that fresh litter input could induce a positive priming effect on soil organic C decomposition, and thus reduced soil C content. However, such a positive priming effect with increasing litter input was not observed in this present study. We observed higher cumulative soil CO_2_-C release in CL0 (0.58 g C) and NL0 (0.39 g C) (S1 Table) compared with litter addition treatments. For example, net C output (C output minus C input) was 0.47 g C in CL1 and 0.35 g C in NL1 (S1 Table), which were lower than that in CL0 and NL0, respectively. Therefore, compared to non-litter addition treatments, litter addition decreased soil C decomposition, which might be related to higher soil C and N concentrations in the larch plantation in this studied site (Table 1). Fontaine et al. [44] recognized that the priming effect depends mainly on the competitions between r-strategist fresh organic carbon decomposers (FOC) and k-strategist SOC decomposers. When nutrients were abundant, probably fresh litter addition stimulated r-strategist FOC decomposers and inhibited k-strategist SOC decomposers [24], thus decreasing SOC mineralization and leading to the negative priming effect. However, the relationship between priming effect and microbial community structure remains to be studied further [45].

We found that soil MBC decreased with increasing litter addition rate while cumulative CO_2_-C release increased. Conversely, Liu and Greaver [6] suggested that soil microbial respiration was positively correlated to soil MBC. The negative response of MBC to litter addition rate could be related to transient N limitations to soil microbial biomass early in the decomposition process [46]. In this study, soil inorganic N decreased in the higher litter input treatments and net N immobilization in litter occurred, thus confirming what has already been reported by Liu et al. [40]. Moreover, there were no differences in soil DOC among different litter addition rate treatments, which might have contributed to the negative response of soil MBC to litter addition. The lower soil MBC but increased CO_2_ release rate in the treatments with higher litter addition rate in our study implied that more soil C substrates might be used for metabolic activities rather than for soil microbial growth [47, 48]. Moreover, competition between r-strategist FOC decomposers (mainly bacteria) and k-strategist SOC decomposers (mainly fungi) is more important to explain the impacts of litter addition rate on litter decomposition and soil respiration rates [44, 45]. Unfortunately, we did not determine biomass and composition of microbial communities of the litter layer in this study. Thus these determinations are needed in the future.

### Effect of N fertilization

We found that N additions inhibited CO_2_ release (17%) though soil inorganic N was higher in NL1 than in CL1, but lower in NL2 than in CL2. The results confirm a recent report that shows that microbial stoichiometry rather than microbial biomass acts as the regulator of soil C and N cycling [41]. This may be because the nutrient in excess of microbial demand is mineralized via waste respiration (for C) or N mineralization [49] to maintain the relatively stable stoichiometry. The results also validate the claim that increased N supply could decrease litter decomposition rate [12, 50–52] due to the suppression of microbial growth and activity [53]. However, some other studies have found that increases in soil available N could increase litter decomposition rates because soil microbes associated with litter decomposition are N limited [54, 55]. In this present study, we observed a significant decline (14%) in soil MBC (Fig 4A and 4B), consistent with the 15% decline in MBC under N addition in both temperate and boreal forests [56]. However, the exact mechanism for the reduction of MBC in N fertilization microcosm is not yet known in our study. There might be two main reasons. First, decreased MBC in N fertilization microcosm are closely related to the decreased soil pH. Demoling et al. [57] showed that soil microbial communities might be altered by decreasing soil pH under N additions. In this study, soil initial pH in N fertilization microcosms (4.21) was lower than that in control microcosms (5.11). Lower soil pH would accelerate soil microbial turnover leading to the reduction in soil microbial biomass [58]. Second, the increase of soil N availability might suppress soil enzyme activity and reduce litter decomposition rate [15, 59]. DeForest et al. [60] indicated that excess NO_3_^−^ altered microbial community function by regulating the activity of enzymes responsible for cellulose and lignin degradation. Zogg et al. [61] also found that anthropogenic NO_3_^−^ could potentially suppress the abundance and activity of lignin degrading fungi. In this study, the suppression of CO_2_ release by N fertilization might account for the higher soil organic C concentration in N addition plots in the larch plantation (Table 1).

In our study, N dynamics in decomposing litter showed a net N immobilization after a transient N release, which is in agreement with a field study by Berg and Staaf [62] who recognized that N dynamics in decomposing litter included three phases of initial leaching, net immobilization and net mineralization. Usually, net N immobilization occurs when litter C:N ratio is higher than 30, but net N mineralization occurs when litter C:N ratio is lower than 30 [63]. In our study, the C:N ratios of the litter in the control and N fertilization microcosms were 90.3 and 82.5, respectively. A greater immobilization of N occurred in the control microcosms, implying that more exogenous N is needed for microbes in the litter collected from the control plots. This might account for the lower soil inorganic N concentration in CL2 as compared to NL2.

There were significant interactions between litter addition rate and N fertilization on CO_2_ release rate, soil MBC and soil DIN concentration, suggesting that the impacts of litter addition rate on soil C balance could be changed by increases in litter N concentration and soil C and N concentrations under N addition. We observed that CO_2_ release rate was significantly lower in NL4 than in CL4, but not for the other litter addition rates, which could induce the occurrence of enhanced soil C sequestration in NL4 but more soil C loss in CL4. In addition, we found that the high peak CO_2_ release in the beginning of litter incubation dropped sharply on the day 14 measurement, which indicated that high microbial activity occurred in the first 7 days. However, respiration rate (Fig 3) decreased sharply in the first two weeks did not mean that microbe and its activity would die or stop in very short time due to the relatively constant levels of MBC during whole experiment (Fig 4). This initial high peak CO_2_ release could be due to the fact that the increasing easily-available organic C after litter input into the soil accelerates the turnover of bacterial biomass (r-strategists FOC decomposers), triggering apparent priming effect [64, 65]. Unfortunately, the initial phase of microbial response (0–14 d) was not fully captured by the weekly measurement. Increased respiration rates at 42 days for the control and at 70 days for the N fertilization could be induced by a real priming effect. When the easily-available C is exhausted, microorganisms that preferentially utilize poorly available substrates (k-strategist SOC decomposers) are stimulated by moribund bacteria and their lysates, which increases soil C decomposition and thus a real priming effect [44, 64]. However, the first CO_2_ measurement was only at day 7 and a long interval between every two measure times, so the cumulative CO_2_ release may be underestimated.

Another caveat is that our laboratory experiment was done under controlled conditions, greatly differing from field conditions. Indeed, inorganic N concentrations were found to increase over incubation time in our study (Fig 5), indicating an accumulation of available N perhaps due to no leaching in the laboratory microcosms. High N concentrations and low C availability (no replenished litter inputs found in forest) in microcosms over incubation time may have inhibited decomposition [66]. The mass loss of larch litter was on average 33% in the microcosms during 270-day incubation, which is equivalent to a period of two years’ growth seasons in the field. Still, our laboratory experiment indicated that increased litter input had a potential to suppress soil organic C loss especially for N fertilization microcosms, which accounts for the higher soil organic C concentration in N addition plots in the larch plantation (Table 1).

**Fig 6:**
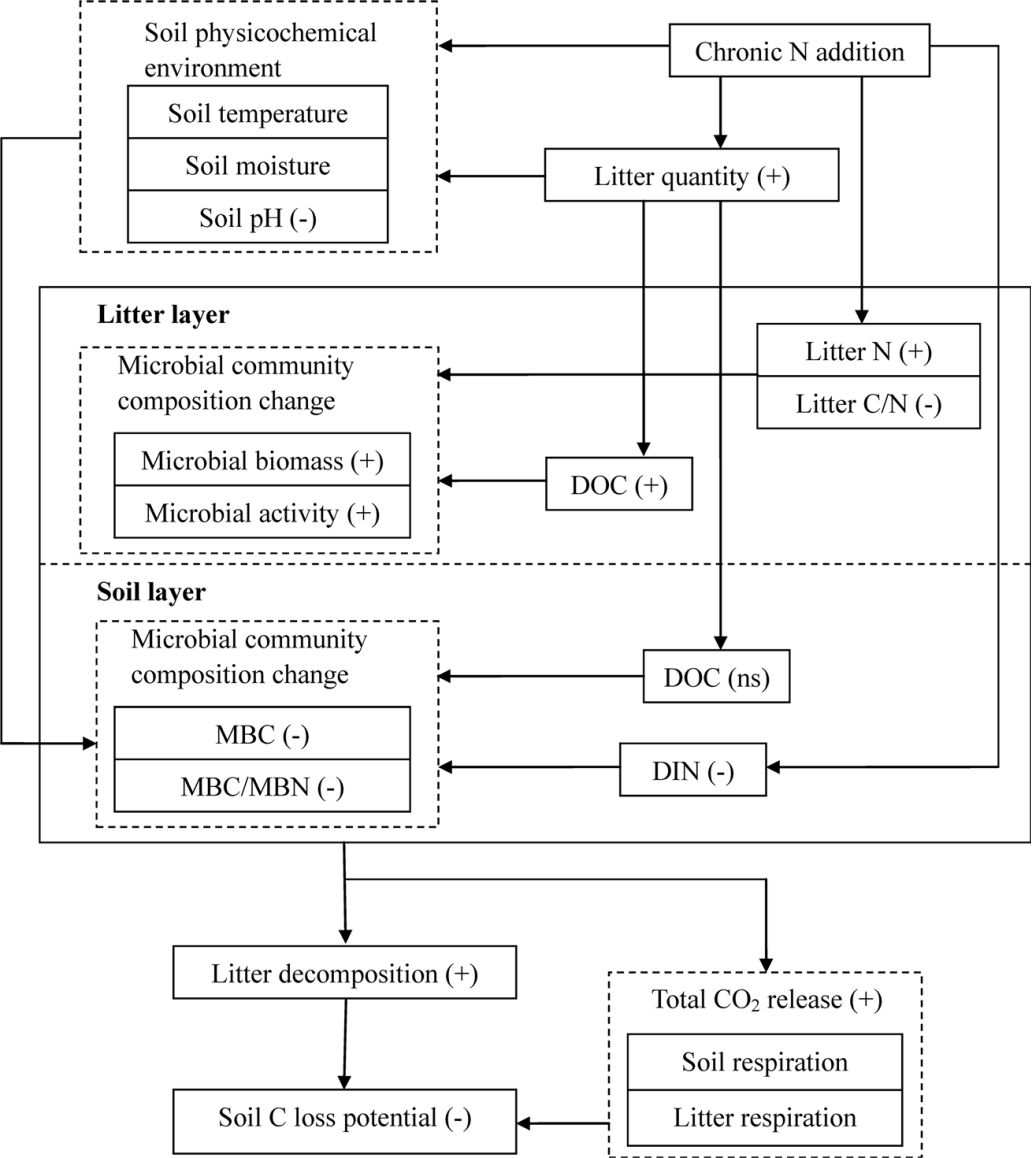
The potential responses of soil carbon and nutrient cycling to changes in litter inputs under N addition. The relationship between the response ratio of each parameter and the quantity of litter inputs is shown in parentheses. “+” indicates a positive correlation; “−” indicates a negative correlation; ns is non-significant. *MBC* microbial biomass carbon; *MBN* microbial biomass nitrogen; *DOC* dissolved organic carbon; *DIN* dissolved inorganic nitrogen.

Taken together, chronic N addition can not only change litter quantity and litter quality controlling litter decomposition, but also alter soil physicochemical environment, such as soil moisture, soil pH, and soil N availability. All these factors play important roles in affecting soil C and nutrient cycling (Fig 6), and are needed to be considered when trying to understand how elevated N deposition affect litter decomposition and SOC sequestration.

## Conclusions

Our results clearly demonstrated that increased litter input had a potential to suppress soil organic C loss although both litter decomposition and CO_2_ release rate increased with increasing input of fresh leaf litter. There were no differences in litter decomposition rates between N fertilization microcosms and control microcosms when litter addition was higher, but CO_2_ release rates were significantly lower in N fertilization microcosms across all litter addition rates due to lower soil microbial biomass. Considering the potential of increased litter input induced by the enhanced N deposition, our results suggest that N addition could help to decrease C losses from soils. In addition, we observed that litter addition did not increase microbial biomass, but litter decomposition rate enhanced, suggesting that microbial biomass is not likely the most important regulator of C mineralization under the condition of higher litter quantity inputs. To better understand the effects of increased litter input on soil C and N dynamics, further studies are needed to elucidate how shifts in litter input affect microbial community structure and stoichiometry and feedback to modify litter decomposition causing stabilization of organic matter in forest soil.

## Supporting Information

S1 Table Carbon budget (g C) of the control and N fertilization microcosms under different litter input quantity treatments after 270 days of incubation. (TIF)

## References

[1] Galloway JN, Townsend AR, Erisman JW, Bekunda M, Cai Z, Freney JR, et al Transformation of the nitrogen cycle: recent trends, questions, and potential solutions. Science. 2008;320:889–892. 10.1126/science.1136674 18487183

[2] Peñuelas J, Sardans J, Rivas-Ubach A, Janssens IA. The human-induced imbalance between C, N and P in Earth's life system. Global Change Biol. 2012;18:3–6.

[3] Pinder RW, Davidson EA, Goodale CL, Greaver TL, Herrick JD, Liu L. Climate change impacts of US reactive nitrogen. Proceedings of the National Academy of Sciences. 2012;109:7671–7675. 10.1073/pnas.1114243109 PMC3356669 22547815

[4] Liu X, Zhang Y, Han W, Tang A, Shen J, Cui Z, et al Enhanced nitrogen deposition over China. Nature. 2013;494:459–462. 10.1038/nature11917 23426264

[5] Tian XF, Hu HW, Ding Q, Song MH, Xu XL, Zheng Y, et al Influence of nitrogen fertilization on soil ammonia oxidizer and denitrifier abundance, microbial biomass, and enzyme activities in an alpine meadow. Biol Fertil Soils. 2014;50:703–713.

[6] Liu L, Greaver TL. A global perspective on belowground carbon dynamics under nitrogen enrichment. Ecol Lett. 2010;13:819–828. 10.1111/j.1461-0248.2010.01482.x 20482580

[7] Adair EC, Reich PB, Hobbie SE, Knops JM. Interactive effects of time, CO_2_, N, and diversity on total belowground carbon allocation and ecosystem carbon storage in a grassland community. Ecosystems. 2009;12:1037–1052.

[8] De Deyn GB, Cornelissen JH, Bardgett RD. Plant functional traits and soil carbon sequestration in contrasting biomes. Ecol Lett. 2008;11:516–531. 10.1111/j.1461-0248.2008.01164.x 18279352

[9] Wang Q, Wang Y, Wang S, He T, Liu L. Fresh carbon and nitrogen inputs alter organic carbon mineralization and microbial community in forest deep soil layers. Soil Biol Biochem. 2014;72:145–151.

[10] Du Y, Guo P, Liu J, Wang C, Yang N, Jiao Z. Different types of nitrogen deposition show variable effects on the soil carbon cycle process of temperate forests. Global Change Biol. 2014;20:3222–3228. 10.1111/gcb.12555 24615991

[11] Frey S, Ollinger S, Nadelhoffer K, Bowden R, Brzostek E, Burton A, et al Chronic nitrogen additions suppress decomposition and sequester soil carbon in temperate forests. Biogeochemistry. 2014;121:305–316.

[12] Janssens I, Dieleman W, Luyssaert S, Subke J-A, Reichstein M, Ceulemans R, et al Reduction of forest soil respiration in response to nitrogen deposition. Nat Geosci. 2010;3:315–322.

[13] Hobbie SE. Contrasting effects of substrate and fertilizer nitrogen on the early stages of litter decomposition. Ecosystems. 2005;8:644–656.

[14] Mao R, Song CC, Zhang XH, Wang XW, Zhang ZH. Response of leaf, sheath and stem nutrient resorption to 7 years of N addition in freshwater wetland of Northeast China. Plant Soil. 2013;364:385–394.

[15] Hobbie SE. Nitrogen effects on decomposition: a five-year experiment in eight temperate sites. Ecology. 2008;89:2633–2644. 18831184 10.1890/07-1119.1

[16] Lovett GM, Arthur MA, Weathers KC, Fitzhugh RD, Templer PH. Nitrogen addition increases carbon storage in soils, but not in trees, in an eastern US deciduous forest. Ecosystems. 2013;16:980–1001.

[17] Knorr M, Frey S, Curtis P. Nitrogen additions and litter decomposition: a meta-analysis. Ecology. 2005;86:3252–3257.

[18] Gärdenäs AI, Ågren GI, Bird JA, Clarholm M, Hallin S, Ineson P, et al Knowledge gaps in soil carbon and nitrogen interactions–from molecular to global scale. Soil Biol Biochem. 2011;43:702–717.

[19] King JS, Kubiske ME, Pregitzer KS, Hendrey GR, McDonald EP, Giardina CP, et al Tropospheric O_3_ compromises net primary production in young stands of trembling aspen, paper birch and sugar maple in response to elevated atmospheric CO_2_ . New Phytol. 2005;168:623–636. 16313645 10.1111/j.1469-8137.2005.01557.x

[20] LeBauer DS, Treseder KK. Nitrogen limitation of net primary productivity in terrestrial ecosystems is globally distributed. Ecology. 2008;89:371–379. 18409427 10.1890/06-2057.1

[21] Chen H, Gurmesa GA, Liu L, Zhang T, Fu S, Liu Z, et al Effects of litter manipulation on litter decomposition in a successional gradients of tropical forests in southern China. PLOS ONE. 2014;9(6):e99018 10.1371/journal.pone.0099018 24901698 PMC4047082

[22] Xiao CW, Guenet B, Zhou Y, Su JQ, Janssens IA. Priming of soil organic matter decomposition scales linearly with microbial biomass response to litter input in steppe vegetation. Oikos. 2015; 124:649–657.

[23] Giardina CP, Binkley D, Ryan MG, Fownes JH, Senock RS. Belowground carbon cycling in a humid tropical forest decreases with fertilization. Oecologia. 2004;139:545–550. 15071736 10.1007/s00442-004-1552-0

[24] Fontaine S, Bardoux G, Abbadie L, Mariotti A. Carbon input to soil may decrease soil carbon content. Ecol Lett. 2004;7:314–320.

[25] Leff JW, Wieder WR, Taylor PG, Townsend AR, Nemergut DR, Grandy AS, et al Experimental litterfall manipulation drives large and rapid changes in soil carbon cycling in a wet tropical forest. Global Change Biol. 2012;18:2969–2679. 10.1111/j.1365-2486.2012.02749.x 24501071

[26] Sayer EJ, Heard MS, Grant HK, Marthews TR, Tanner EV. Soil carbon release enhanced by increased tropical forest litterfall. Nat Clim Change. 2011;1:304–307.

[27] Hu YL, Zeng DH, Liu YX, Zhang YL, Chen ZH, Wang ZQ. Responses of soil chemical and biological properties to nitrogen addition in a Dahurian larch plantation in Northeast China. Plant Soil. 2010;333:81–92.

[28] Jia S, Wang Z, Li X, Sun Y, Zhang X, Liang A. N fertilization affects on soil respiration, microbial biomass and root respiration in *Larix gmelinii* and *Fraxinus mandshurica* plantations in China. Plant Soil. 2010;333:325–336.

[29] Wang Z, Guo D, Wang X, Gu J, Mei L. Fine root architecture, morphology, and biomass of different branch orders of two Chinese temperate tree species. Plant Soil. 2006;288:155–171.

[30] IUSS Working Group W. World reference base for soil resources World Soil Resources Report. 2006;103 FAO, Rome.

[31] Nelson DW, Sommers LE. Total carbon, organic carbon, and organic matter In: Methods of soil analysis: Part 3 Chemical methods. Soil Science Society of America, Inc. and American Society of Agronomy, Inc. Madison, Wisconsin, USA 1996:961–1010.

[32] Bremner J. Nitrogen–total In: Methods of soil analysis: Part 3 Chemical methods. Soil Science Society of America, Inc. and American Society of Agronomy, Inc. Madison, Wisconsin, USA 1996:1085–1121.

[33] Iiyama K, Wallis AF. Determination of lignin in herbaceous plants by an improved acetyl bromide procedure. J Sci Food Agr. 1990;51:145–161.

[34] Updegraff DM. Semimicro determination of cellulose inbiological materials. Anal Biochem. 1969;32:420–424. 5361396 10.1016/s0003-2697(69)80009-6

[35] Waterman PG, Mole S. Analysis of phenolic plant metabolites: Blackwell Scientific; 1994.

[36] Vance E, Brookes P, Jenkinson D. An extraction method for measuring soil microbial biomass C. Soil Biol Biochem. 1987;19:703–707.

[37] Brookes PC, Landman A, Pruden G, Jenkinson D. Chloroform fumigation and the release of soil nitrogen: a rapid direct extraction method to measure microbial biomass nitrogen in soil. Soil Biol Biochem. 1985;17:837–842.

[38] Wu J, Liu Z, Wang X, Sun Y, Zhou L, Lin Y, et al Effects of understory removal and tree girdling on soil microbial community composition and litter decomposition in two Eucalyptus plantations in South China. Funct Ecol. 2011;25:921–931.

[39] Robertson GP, Wedin D, Groffman P, Blair J, Holland E, Nedelhoffer K, et al Soil carbon and nitrogen availability. Nitrogen mineralization, nitrification and soil respiration potentials. Standard soil methods for long-term ecological research Oxford University Press, New York 1999:258–271.

[40] Liu LL, King JS, Booker FL, Giardina CP, Lee Allen H, Hu SJ. Enhanced litter input rather than changes in litter chemistry drive soil carbon and nitrogen cycles under elevated CO_2_: a microcosm study. Global Change Biol. 2009;15:441–453.

[41] Buchkowski RW, Schmitz OJ, Bradford MA. Microbial stoichiometry overrides biomass as a regulator of soil carbon and nitrogen cycling. Ecology. 2015;96:1139–1149. 26230033 10.1890/14-1327.1

[42] Drake JE, Darby BA, Giasson MA, Kramer MA, Phillips RP, Finzi AC. Stoichiometry constrains microbial response to root exudation-insights from a model and a field experiment in a temperate forest. Biogeosciences. 2013;10:821–838.

[43] Schimel JP, Weintraub MN. The implications of exoenzyme activity on microbial carbon and nitrogen limitation in soil: a theoretical model. Soil Biol Biochem. 2003;35:549–563.

[44] Fontaine S, Mariotti A, Abbadie L. The priming effect of organic matter: a question of microbial competition? Soil Biol Biochem. 2003;35:837–843.

[45] Kuzyakov Y. Priming effects: interactions between living and dead organic matter. Soil Biol Biochem. 2010;42:1363–1371.

[46] Berg B, Laskowski R. Decomposers: soil microorganisms and animals. Adv Ecol Res. 2005;38:73–100.

[47] De Nobili M, Contin M, Mondini C, Brookes P. Soil microbial biomass is triggered into activity by trace amounts of substrate. Soil Biol Biochem. 2001;33:1163–1170.

[48] Kuzyakov Y, Bol R. Sources and mechanisms of priming effect induced in two grassland soils amended with slurry and sugar. Soil Biol Biochem. 2006;38:747–758.

[49] Sterner RW, Elser JJ. Ecological stoichiometry: the biology of elements from molecules to the biosphere: Princeton University Press; 2002.

[50] Carreiro M, Sinsabaugh R, Repert D, Parkhurst D. Microbial enzyme shifts explain litter decay responses to simulated nitrogen deposition. Ecology. 2000;81:2359–2365.

[51] Coûteaux M-M, Bottner P, Berg B. Litter decomposition, climate and liter quality. Trends Ecol Evol. 1995;10:63–66. 21236954 10.1016/S0169-5347(00)88978-8

[52] Prescott C. Does nitrogen availability control rates of litter decomposition in forests? Nutrient Uptake and Cycling in Forest Ecosystems: Springer Netherlands; 1995 p. 83–88.

[53] Vitousek PM, Aber JD, Howarth RW, Likens GE, Matson PA, Schindler DW, et al Human alteration of the global nitrogen cycle: sources and consequences. Ecol Appl. 1997;7:737–750.

[54] McClaugherty CA, Pastor J, Aber JD, Melillo JM. Forest litter decomposition in relation to soil nitrogen dynamics and litter quality. Ecology. 1985:266–275.

[55] Melillo JM, Aber JD, Muratore JF. Nitrogen and lignin control of hardwood leaf litter decomposition dynamics. Ecology. 1982;63:621–626.

[56] Treseder KK. Nitrogen additions and microbial biomass: A meta-analysis of ecosystem studies. Ecol lett. 2008;11:1111–1120. 10.1111/j.1461-0248.2008.01230.x 18673384

[57] Demoling F, Ola Nilsson L, Bååth E. Bacterial and fungal response to nitrogen fertilization in three coniferous forest soils. Soil Biol Biochem. 2008;40:370–379.

[58] Wardle DA. Controls of temporal variability of the soil microbial biomass: A global-scale synthesis. Soil Biol Biochem. 1998;30:1627–1637.

[59] Pregitzer KS, Burton AJ, Zak DR, Talhelm AF. Simulated chronic nitrogen deposition increases carbon storage in Northern Temperate forests. Global Change Biol. 2008;14:142–153.

[60] DeForest JL, Zak DR, Pregitzer KS, Burton A J. Atmospheric nitrate deposition, microbial community composition, and enzyme activity in northern Hardwood forests. Soil Sci Soc Am J 2004; 68:132–138

[61] Zogg GP, Zak DR, Pregitzer KS, Burton A J. Microbial immobilization and the retention of anthropogenic nitrate in a northern hardwood forest. Ecology. 2000:1858–1866.

[62] Berg B, Staaf H. Decomposition rate and chemical changes of Scots pine needle litter. II. Influence of chemical composition. Ecol Bull. 1980:373–390.

[63] Wedin DA, editor Nitrogen availability, plant-soil feedbacks and grassland stability. Proceedings of the VI International Rangeland Congress on People and Rangelands Building the Future Townsville, Australia; 1999.

[64] Blagodatskaya EV, Blagodatsky SA, Anderson TH, Kuzyakov Y. Priming effects in Chernozem induced by glucose and N in relation to microbial growth strategies. Appl Soil Ecol. 2007;37:95–105.

[65] Nottingham AT, Griffiths H, Chamberlain PM, Stott AW, Tanner EVJ. Soil priming by sugar and leaf-litter substrates: A link to microbial groups. Appl Soil Ecol. 2009;42:183–190.

[66] Fogg K. The effect of added nitrogen on the rate of organic matter decomposition. Biol Rev. 1988;63:433–472.

